# Oligo-Alginate with Low Molecular Mass Improves Growth and Physiological Activity of *Eucomis autumnalis* under Salinity Stress

**DOI:** 10.3390/molecules23040812

**Published:** 2018-04-02

**Authors:** Piotr Salachna, Monika Grzeszczuk, Edward Meller, Marcin Soból

**Affiliations:** 1 Department of Horticulture, West Pomeranian University of Technology, 3 Papieża Pawła VI Str., 71-459 Szczecin, Poland; monika.grzeszczuk@zut.edu.pl; 2 Department of Soil Science, Grassland Management and Environmental Chemistry, West Pomeranian University of Technology, Słowackiego 17 Str., 71-434 Szczecin, Poland; edward.meller@zut.edu.pl; 3 Center of Bioimmobilisation and Innovative Packaging Materials, West Pomeranian University of Technology, 35 Janickiego Str., 71-270 Szczecin, Poland; marcin.sobol@zut.edu.pl

**Keywords:** oligosaccharides, degree of polymerization, NaCl, plant growth promoter

## Abstract

Biopolymers have become increasingly popular as biostimulators of plant growth. One of them, oligo-alginate, is a molecule that regulates plant biological processes and may be used in horticultural practice as a plant growth regulator. Biostimulators are mainly used to improve plant tolerance to abiotic stresses, including salinity. The aim of the study was to assess the effects of salinity and oligo-alginate of various molecular masses on the growth and physiological activity of *Eucomis autumnalis*. The species is an ornamental and medicinal plant that has been used for a long time in the traditional medicine of South Africa. The bulbs of *E. autumnalis* were coated using depolymerized sodium alginate of molecular mass 32,000; 42,000, and 64,000 g mol^−1^. All of these oligo-alginates fractions stimulated plant growth, and the effect was the strongest for the fraction of 32,000 g mol^−1^. This fraction was then selected for the second stage of the study, when plants were exposed to salt stress evoked by the presence of 100 mM NaCl. We found that the oligo-alginate coating mitigated the negative effects of salinity. Plants treated with the oligomer and watered with NaCl showed smaller reduction in the weight of the above-ground parts and bulbs, pigment content and antioxidant activity as compared with those not treated with the oligo-alginate. The study demonstrated for the first time that low molecular mass oligo-alginate may be used as plant biostimulator that limits negative effects of salinity in *E. autumnalis*.

## 1. Introduction

Biostimulators are enjoying considerable attention as chemical or microbial agents that improve plant growth and yield quality [1,2]. The worldwide market for biostimulators is expected to be worth over two billion dollars in 2018 [3]. Algae-derived polysaccharides are popular biostimulators with multidirectional effects in plants [4]. They are biodegradable, biocompatible, non-toxic, biologically reactive, and cheap [5]. Of particular interest are alginates—natural polysaccharide copolymers composed of two types of monomers: β-d-mannaric acid and α-l-guluronic acid [6]. Alginate is a well-known polysaccharide obtained from marine algae, mainly brown algae (Phaeophyceae) or produced extracellularly by some bacteria [7]. The literature review shows that sodium alginate in depolymerized form stimulates seed germination [8], plant growth and development [9,10,11], affects photosynthesis [12], ion uptake and transport [13,14], and increases the content of assimilation pigments [15], proteins [16], and secondary metabolites such as artemisinin [12], morphine, codeine [17] or essential oils [18,19]. In addition, sodium alginate is effective in inhibiting certain plant diseases [20], enhances plant tolerance to drought [21] and alleviates toxic effects of heavy metals [22]. Sodium alginate has been used for many years in horticulture for producing biopolymeric seed coatings, sometimes supplemented with fertilizers and plant protection products [23]. It is also successfully used for coating fruits and vegetables to prolong their shelf life [24]. Sodium alginate is useful for encapsulation of in vitro somatic embryos and cultured shoot tips to develop synthetic seeds of many plant species [25,26].

Bioactive oligosaccharides of a specific structure and size act as signal molecules [27,28] that may control plants’ growth [29] and basal metabolism [30] by regulating their gene expression [31]. Research shows that depolymerized sodium alginate is a more effective plant growth promoter than a non-polymerized form [12,13,32]. This might be due to its low molecular mass, which is one of the most important factors affecting biological activity of many chemically modified polysaccharides [33,34,35].

Biostimulators, including polysaccharides, are often applied in the form of spraying or watering solutions, which requires a lot of work and large amounts of water. An answer to this problem may be the use of hydrogel coatings containing the biostimulators [36]. Such coatings are the result of a polyelectrolyte complex formation by interaction of anionic functional groups of the polyelectrolyte with metal cations, or of a reaction at the interface of aqueous solutions of polyelectrolytes with functional groups of opposite charges [37]. Polysaccharides applied via coatings may positively affect plant growth and flowering [38] and stimulate root system development [39]. Specific effects depend on the components used [40].

*Eucomis autumnalis* (Mill.) Chitt (Asparagaceae formerly Hyacinthaceae) is an ornamental plant, highly popular due to its extremely original, decorative and durable inflorescences. It is grown as a pot plant and for cut flowers [41,42]. The plants may be grown in gardens and urban green areas, but their sensitivity to excess soil salinity should be taken into account [43]. *E. autumnalis* is the most important medical plant, used for a long time in traditional South African medicine [44]. Bulbs, leaves and roots of *E. autumnalis* show multidirectional pharmacological effects, including antioxidant, anti-inflammatory, bactericidal, fungicidal and cytostatic activity [45,46]. As the bulbs have been in very high demand for pharmacological purposes, the species is now threatened with extinction [47]. Therefore, the aim of many scientific studies is to develop new solutions aimed at providing the plants with the most favorable growth conditions [48,49].

Salinity is one of the crucial abiotic stresses limiting plant growth [50]. Exceeding the plant tolerance threshold for salt triggers numerous adverse changes in plant growth and development [51]. Negative effects of salt stress include mainly the accumulation and toxicity of Na^+^ and Cl^−^ ions, disturbance of ionic equilibrium, dysfunction of plasma membranes, changes in metabolism and enzymatic activity, and limitation of photosynthesis and respiration intensity [52]. Moreover, salinity limits growth and flowering, induces wilting, drying and falling of leaves, disrupts water management and generates oxidative stress [53]. To improve plant tolerance to unfavorable factors, breeders may use anti-stress substances, including e.g., polysaccharides [54] and their derivatives such as chitosan-PVA [55], sulfated chitooligosaccharides [56] or gamma irradiated carrageenan [57]. So far, data are missing on the effects of sodium alginate derivatives on the growth of plants exposed to salt stress. 

This study verified a research hypothesis that sodium alginate of various molecular masses, and therefore with different physical and chemical properties, applied in the form of bulb coating, affects the growth of *E. autumnalis* to varying degrees. We also made the first attempt at alleviating the effects of salt stress in *E. autumnalis* by treating the plants with a selected fraction of sodium alginate with the strongest biostimulatory effect.

## 2. Results and Discussion

### 2.1. Fourier Transform Infra Red (FTIR)

**Figure 1:**
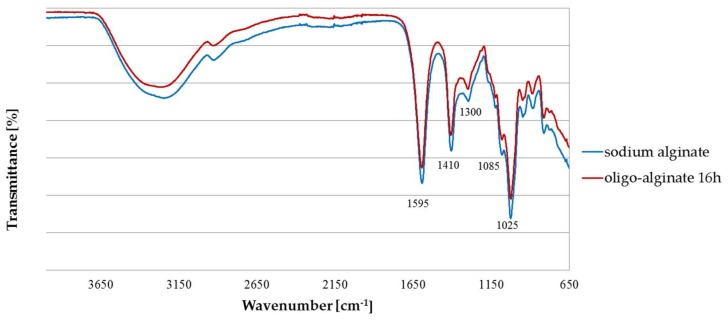
Fourier transform infra red (FTIR) spectra of sodium alginate and oligo-alginate obtained after 16 h hydrolysis.

Spectroscopic analyses were performed in order to confirm whether alginates undergo any chemical modification after the depolymerization process. Sodium alginate and oligo-alginate spectrum (Figure 1) showed bands at ~1595 cm^−1^ and ~1410 cm^−1^, which is given by the antisymmetric and symmetric COO^−^ stretch, respectively. Band at ~1300 cm^−1^ is assign to the skeletal vibration [58]. Peak at ~1085 cm^−1^ is attributed to C–O–C stretching and at ~1025 cm^−1^ to the OH bending of guluronate [59]. 

### 2.2. Effects of Oligo-Alginates with Different Molecular Mass on Growth Attributes of Eucomis autumnalis

**Figure 2:**
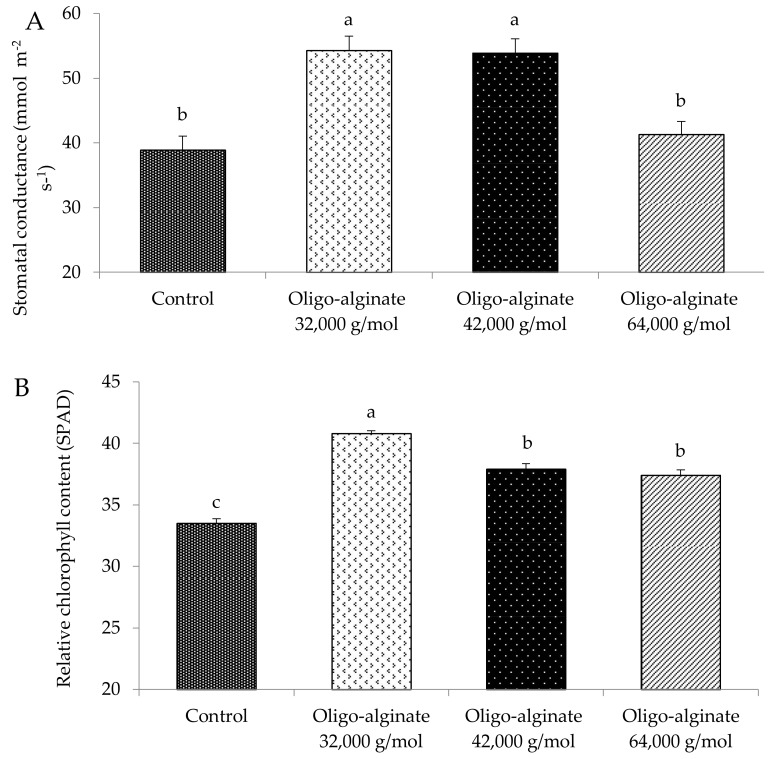
Effect of oligo-alginate with different molecular mass on stomatal conductance (**A**) and the relative chlorophyll content (**B**) of *Eucomis autumnalis*. Data are presented as means (±standard errors) and bars with different letters in each graph are significantly different by Tukey test (*p* ≤ 0.05).

In the first year, we assessed the effects of coating *E. autumnalis* bulbs in oligo-alginates of different molecular mass on plant morphological features and physiological parameters (Table 1, Figure 2). Compared with control plants, the plants treated with all fractions of oligo-alginates flowered earlier (on average by 5.5 days), and had longer leaves (33.2%) and inflorescences (33.6%). Molecular mass of the oligomers did not affect these variables. The oligo-alginate of molecular mass 32,000 g mol^−1^ caused the highest increase in plant height (30.8%), plant width (28.6%), florets number per inflorescence (29.3%) and the relative chlorophyll content (21.8%) in comparison with control plants. Moreover, plants treated with 32,000 and 42,000 g mol^−1^ oligo-alginates had greater than controls stomatal conductance, by 39.6% and 38.6%, respectively. The oligomers did not affect the number of produced leaves and inflorescences. These results corroborated those from other studies in which depolymerized sodium alginate positively influenced morphological features of plants depending on their degree of polymerization. The irradiated alginate with molecular mass below 10^4^ considerably stimulated growth of rice and peanut through a hydroponic solution [9]. Media supplemented with irradiated alginate of molecular mass approximately 1.43 × 10^4^ Da positively affected shoot height, root length and fresh weight of in vitro propagated ornamental plants *Chrysanthemum* × *grandiflorum*, *Eustoma grandiflorum* and *Limonium latifolium* [10]. Irradiated alginate of molecular mass within 1–3 kDa most effectively stimulated growth and development of barley and soybean [11]. Research showed that oligosaccharides in higher plants shorten the cell cycle, stimulate cell elongation and cell division, enhance mitotic indexes as well as decrease chromosomal aberration frequency [22,60]. Stronger effects of low molecular mass oligosaccharides may result from faster and easier penetration of their molecules into plant tissues and individual cells [61]. 

### 2.3. Effects of Oligo-Alginate and Salinity Stress on Growth and Physiological Attributes of Eucomis autumnalis

**Figure 3:**
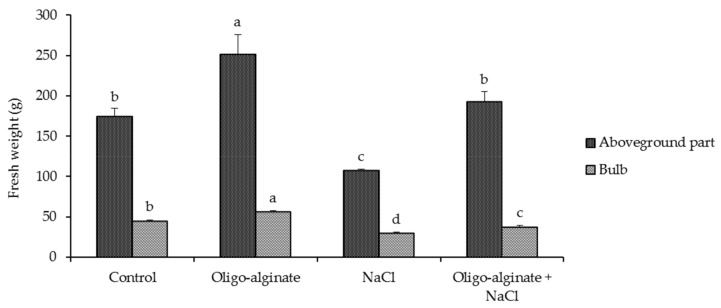
Effect of oligo-alginate and NaCl on fresh weight of above-ground part and bulb of *Eucomis autumnalis*. Data are presented as means (±standard errors) and bars with different letters in each graph are significantly different by Tukey Test (*p* ≤ 0.05).

The next phase of the study focused on the response of plants treated with sodium-alginate to salt stress. The bulbs were coated in oligo-alginate of molecular mass 32,000 g mol^−1^ that most strongly stimulated plant height, number of florets per inflorescence and the leaf greenness index Soil and Plant Analysis Developmen SPAD (Table 1, Figure 2). The effects of the oligomer and salinity on plant growth were estimated based on fresh biomass increase, as this parameter is useful in determining the quality of ornamental plants (Figure 3). Plants treated with oligo-alginate had considerably greater fresh weight of the above-ground parts and bulbs by 46.5% and 26.0%, respectively, as compared with controls. The increased biomass of the plants treated with the degraded alginate may be due to enhanced uptake of water and minerals by roots, and thus higher levels of nutrients in plant tissues, as reported for *Eucalyptus citriodora* [13], *Artemisia annua* [14] and *Cymbopogon flexuosus* [16]. 

The fresh weight of the above-ground parts and bulbs in salt-treated plants dropped markedly, by 37.8% and 34.5%, respectively, in comparison with non-treated plants (Figure 3). The adverse effect of salt stress on the biomass of leaves, inflorescences or bulbs was also observed in other geophytes [62,63]. Salt stress inhibits mitoses and elongation growth of cells and consequently reduces fresh and dry weight, especially of the above-ground parts [64].

In this study, oligo-alginate treatment effectively protected plants from biomass loss caused by exposure to salinity. Salt-induced reduction in fresh weight of the above-ground parts and bulbs was smaller in the plants treated with oligo-alginate vs. those treated with NaCl alone (Figure 3). Oligosaccharides may facilitate plant survival in unfavorable environments by interacting with cellular membrane receptors in a hormone-like manner and affecting gene regulation [65]. Oligosaccharides may increase plant stress tolerance by inducing the enzymes of the antioxidant system and stimulating synthesis of phenolic compounds [66,67]. Stress alleviating activity of the oligomers depends largely on their physical and chemical properties, including the degree of polymerization [34] and acetylation [68].

**Figure 4:**
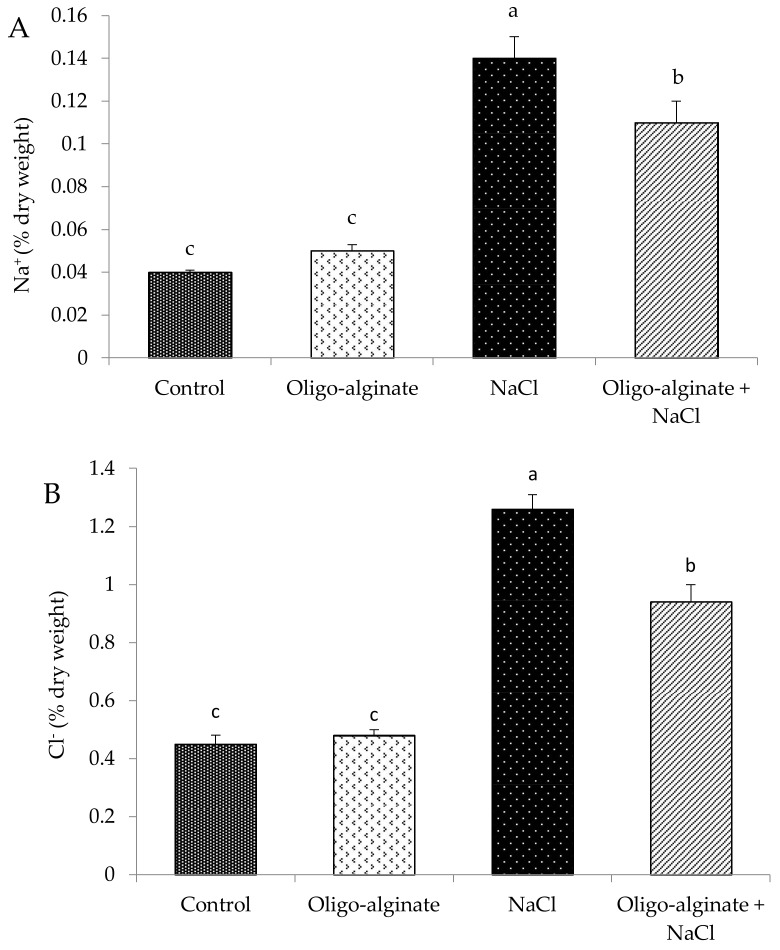
Effect of oligo-alginate and NaCl on the Na^+^ (**A**) and Cl^−^ (**B**) ion concentration in leaves of *Eucomis autumnalis*. Data are presented as means (±standard errors) and bars with different letters in each graph are significantly different by Tukey Test (*p* ≤ 0.05).

Leaves of the plants exposed to salinity accumulated significantly more sodium and chlorine than the control plants by 3.5- and 2.8-fold, respectively (Figure 4). The same trend was also reported for other bulbous plants [64,69]. Coating the bulbs in oligo-alginate curbed the accumulation of sodium and chlorine in salt exposed plants. The mechanisms of tolerating increased concentration of salt involve a synthesis of compatible metabolites that protect the structure of proteins and membranes (amino acids, amines, carbohydrates and polyols) [51,70]. 

The content of photosynthetic pigments provides a lot of information on how the biostimulators work under salt stress [71]. Quantitative proportions of chlorophyll *a*, *b* and carotenoids are also important for controlling photosynthesis intensity [72]. Our study confirmed positive effects of bulb coating with the oligo-alginate on leaf content of photosynthetic pigments (Table 2). The plants treated with the oligo-alginate contained significantly more chlorophyll *a* (by 13.8%) and chlorophyll *a* + *b* (by 13.8%) than the non-treated ones. Enhanced content of leaf pigments in oligo-alginate treated plants might improve photosynthetic efficiency, which is usually associated with improved uptake of minerals and finally leads to greater biomass production. Foliar application of oligo-alginate increased the content of photosynthetic pigments in the leaves of other medicinal plants [15,16]. This was probably due to a positive effect of the oligomer on the efficiency of the photosynthetic apparatus. Oligo-alginate has been reported to improve net photosynthetic rate, stomatal conductance, internal CO_2_ concentration, as well as the activity of carbonic anhydrase—an enzyme transferring carbon dioxide to RuBisCO during photosynthesis [11,12,13].

Plants exposed in our study to salt stress had 21.1% lower content of chlorophyll *a*, 12.1% chlorophyll *b*, 18.6% chlorophyll *a* + *b* and 9.9% carotenoids than the control ones. They also had reduced chlorophyll *a* to chlorophyll *b* ratio and chlorophyll *a + b* to carotenoids ratio. Oligo-alginate treatment in salinity-exposed plants significantly limited the loss of chlorophyll *a* and chlorophyll a + *b*, and slightly reduced the decrease in carotenoids and chlorophyll *a* to chlorophyll *b* ratio vs. plants non-treated with the oligomer (Table 2). Excessive amounts of salt accumulated in chloroplasts exert a direct toxic effect on photosynthesis through destabilization of protein complexes and destruction of photosynthetic pigments [52,53]. A drop in the chlorophyll *a + b* to carotenoids ratio may be a sign of oxidative stress and also shows that a plant has activated the mechanisms of antioxidant defense by increasing the content of carotenoids responsible for protecting photosytems against reactive oxygen species [71,72,73].

Apart from yield increase, the positive effects of oligosaccharide application often include improvements in yield quality, including secondary metabolites [15,17,18]. Therefore, we decided to determine total polyphenol content (TPC) in the leaves. We also assessed content of l-ascorbic acid and plant antioxidant activity (DPPH) to better understand the response of the antioxidant system to oligo-alginate and NaCl treatment (Table 3). 

The highest TPC, l–ascorbic acid and antioxidant activities were observed in the plants grown from the bulbs coated with oligo-alginate. These variables were the lowest in the control plants. Salt stress induced a significant increase in TPC, l-ascorbic acid and a stimulate in antioxidant activity vs. plants non-treated with NaCl. Application of oligo-alginate in NaCl treated plants did not change their total polyphenol content and slightly enhanced content of l-ascorbic acid and antioxidant activity. The positive effect of oligo-alginate on limiting the drop in l-ascorbic acid and antioxidant activity under salt stress indicates protective properties of this agent. Sodium alginate derivatives change plant metabolism and affect biosynthesis of various substances, including active constituents [12,15,18], but the mechanism remains unclear. 

The results discussed above demonstrate that sodium alginate derivatives favorably affect morphological features and physiological and biochemical parameters of *Eucomis autumnalis* and improve the medicinal value of the plant. The plants treated with oligo-alginate achieved markedly higher biomass, pigment content, total polyphenol content and antioxidant activity. Polysaccharides and their derivatives stimulate or inhibit specific enzymes or enzymatic pathways involved in metabolic processes [27,31,74]. They may also act inside plant cells and control their hormonal activity [75]. Sodium alginate oligomers may stimulate plant growth by enhancing expression of selected genes. For example, alginate oligosaccharides induced expression of an auxin-related gene in rice, which changed auxin content in roots and promoted root formation and elongation [76]. Therefore, future studies evaluating the response of *Eucomis autumnalis* to oligo-alginate could include examination of genetic factors affecting plant growth and resistance to various microorganisms. 

Our study demonstrated that oligo-alginate alleviates negative effects of salt stress in *Eucomis autumnalis*. We hypothesize that its mechanism of action consisted in limiting the accumulation of harmful Cl^−^ and Na^+^ ions in leaf tissues, which might finally result in reduced biomass loss and smaller decrease in the content of assimilation pigments. Interestingly, salt-exposed plants treated with oligo-alginate had higher leaf levels of l-ascorbic acid and higher antioxidant activity (DPPH), which may suggest activation of stress adaptation mechanisms. Plants experiencing salt stress remove reactive oxygen species (ROS) via non-enzymatic mechanisms, such as scavenging with ascorbic acid, and by increasing production of antioxidant enzymes [52,53]. Oligo-alginate may stimulate ROS detoxifying enzymes and enhance activity of isozymes responsible for stress protection. This hypothesis was confirmed by Zhang et al. [77], who reported that algino-oligosaccharides induced the activity of phenylalanine ammonia lyase (PAL), peroxidase (POD) and catalase (CAT) in rice cells and protected plants against pathogens. Changes in the activity of antioxidant enzymes may most probably determine the anti-stress effect of oligo-alginate in plants, but this requires further research.

## 3. Materials and Methods

### 3.1. Plant Materials and Growth Conditions

The study was conducted in the years 2014–2015 in a plastic tunnel located in the area of West Pomeranian University of Technology in Szczecin (53°25′ N, 14°32′ E; 25 m above mean sea level). Plant material included the bulbs of *E. autumnalis*, with a perimeter of 14–16 cm, purchased in the Netherlands. The bulbs were planted each year in mid-April into PVC pots (18 cm) filled with peat substrate enriched with 3 g dm^−3^ of a multicomponent fertilizer Hydrocomplex (Yara International ASA, Oslo, Norway) containing (in % mass): N-NO_3_ (5), N-NH_4_ (7), P_2_O_5_ (11), K_2_O (18), MgO (2.7), S (8), B (0.015), Fe (0.2), Mn (0.02), and Zn (0.02). The peat substrate of pH 5.7 contained 0.37 g NaCl dm^−3^ and also (in mg dm^−3^): N-NO_3_ (10), P (37), K (22), Ca (1980), Mg (122), and Cl (16). The plants were cultivated until mid-September under natural photoperiod and watered on average three times a week with tap water of the following composition (in mg dm^−3^): N-NO_3_ (1.53), P (1.50), K (6.2), Ca (97.4), Mg (16.6), Na (24.0), Cl (24.0), Cu (0.62), Zn (0.42), Fe (1.3), HCO_3_ (194), pH 6.4, and electrolytic conductivity 0.64 mS cm^−1^. Mean air temperature (°C) and relative humidity (%) in the plastic tunnel in 2014 were as follows: April 15.1/71.4, May 18.9/76.9, June 19.7/70.6, July 24.1/75.0, August 19.6/78.5, September 18.2/80.4, and in 2015: April 13.6/73.3, May 15.4/60.8, June 18.1/73.3, July 20.8/68.3, August 24.1/59.5, and September 17.4/70.6.

### 3.2. Preparation and Determination the Molecular Mass of Oligo-Alginates

Oligo-alginates were prepared by acid hydrolysis. Twenty grams of starting sodium alginate from algae (Sigma-Aldrich, Poznań, Poland) was dissolved in 800 mL of deionized water at 70 °C. Concentrated HCl (Chempur, Piekary Śląskie, Poland) was added to obtain final concentration of 0.2 M. Then the solution was incubated for 4, 8 or 16 h while stirring. Starting sodium alginate had molecular mass of 325,000 g mol^−1^, which decreased during 4, 8 and 16 hours of hydrolysis to 64,000 g mol^−1^, 42,000 g mol^−1^ and 32,000 g mol^−1^, respectively. Following hydrolysis samples were cooled to room temperature, neutralized with NaOH to obtain pH 7 and partially evaporated using vacuum evaporator (RVO 200A, INGOS, Praha, Czech Republic). High Performance Size Exclusion Chromatography (HPSEC) was utilized to define molecular masses of starting and depolymerized sodium alginates. High-pressure dosing pump S 1000, refractive index detector S 2300 and a 20 µL sample loop (Knauer, Berlin, Germany). Separation was performed using SUPREMA 10,000 Å 10 µm column (PSS, Mainz, Germany) eluted with 0.5 M NaCl at a flow rate of 1 mL min^−1^ in room temperature. Relative molecular mass was determined using pullulans with narrow polydispersity (PSS, Mainz, Germany) as standards for calibration. Peak molecular mass of pullulan standards was as follow: 342; 1080; 6100; 9600; 21,100; 47,100; 107,000; 194,000; 344,000; and 708,000 g mol^−1^.

### 3.3. Fourier Transform Infra Red (FTIR) Analysis

Attenuated total reflectance Fourier transform infrared (ATR-FTIR) spectra were recorded using Spectrum 100 spectrometer, equipped with a diamond ATR cystal (Perkin Elmer Spectrophotometer, Spectrum 100, Waltham, MA, USA), with a resolution of 4 cm^−1^ in the 4000–650 cm^−1^ wavenumber range.

### 3.4. Experiments

#### 3.4.1. Effects of Oligo-Alginates with Different Molecular Mass on Growth of *Eucomis autumnalis*

In the first year of the study *E. autumnalis* bulbs were coated with the oligo-alginates of different molecular weight (32,000; 42,000 and 64,000 g mol^−1^). The controls were non-coated bulbs. The coating method was based on polyelectrolyte complexes [36]. The bulbs were soaked for 30 s in 1% aqueous solutions of oligo-alginates. The 40 bulbs were coated per treatment, ten per each repetition. After drying, the bulbs were planted individually into pots, which were then placed on tables in the plastic tunnel. 

##### Determination of Growth Parameters

We determined the number of days from planting the bulbs to the beginning of flowering marked by the appearance of the first flowers. At this phase, 10 plants from each repetition were randomly selected to measure their height and width, the number of leaves and length of the longest leaf, and the number and length of inflorescences. Moreover, leaf greenness index SPAD (Soil and Plant Analysis Development) was measured with Chlorophyll Meter SPAD 502 (Minolta, Osaka, Japan) and leaf stomatal conductance was assessed with SC1 porometer (Dekagon Devices, Pullman, WA, USA). The measurements involved two fully developed leaves of five randomly selected plants from each repetition. They were repeated three times for each leaf and mean value was calculated. When the flowering was over, the number of flowers was assessed based on the number of inflorescence pedicels.

#### 3.4.2. Effect of Oligo-Alginate and Salt Stress on Plant Growth and Physiological Activity

In the second year of the study, the following experimental variants were tested: (I) control, non-treated plants; (II) bulbs coated with oligo-alginate before planting; (III) plants watered with 100 mM NaCl; (IV) bulbs coated with oligo-alginate before planting and plants watered with 100 mM NaCl. The concentration of NaCl solution was determined based on earlier studies that demonstrated considerable biomass decrease in the presence of 100 mM NaCl. Each variant involved 40 bulbs, ten per repetition. Considering the results from the first year, the oligo-alginate of molecular mass 32,000 g mol^−1^ was selected for further bulb coating studies. The coated bulbs were planted individually into pots. After 30 days from planting, we started to water the plants with 100 mM NaCl (Chempur, Piekary Śląskie, Poland) solution. The plants were watered twice a week, for eight weeks, with 100 mL of the solution per plant per treatment. The plants in the other variants were treated with water. When the salt treatment ended, the plants were removed from the pots and fresh weight of their above-ground parts and bulbs was assessed. Fresh leaves were used for analyses.

##### Determination of Photosynthetic Pigments

Leaf pigment content was determined using a spectrophotometer [78]. Fresh plant material was extracted with 80% acetone. Absorption of the extract was measured with spectrophotometer (Helios Gamma, Thermo Spectronic, Cambridge, UK) at 441, 646, 652, and 663 nm. The following formulas were used in the calculations:

chlorophyll *a* (mg kg^−1^ FW) = (12.21 × E_663_ − 2.81 × E_646_) × (V/1000 × m),

chlorophyll *b* (mg kg^−1^ FW) = (20.13 × E_646_ − 5.03 × E_663_) × (V /1000 × m),

carotenoids (mg kg^−1^ FW) = [(1000 × E_441_) − 3.27 × (12.21 × E_663_ − 2.81 × E_646_) − 104 × (20.13 × E_646_ − 5.03 × E_663_) ] × [V/1000 × (m × 229)],

where

E—extinction at specific wavelength,
V—volume of a volumetric flask [cm^3^],
m—sample weight in g.

##### Determination of of l-Ascorbic Acid

The content of vitamin C as L-ascorbic acid was determined by the Tillmans method, based on the color reaction of ascorbic acid with 2,6-dichloroindophenol solution [79].

##### Determination of Total Polyphenols

Phenolic compounds were extracted from leaves as described before [80]. Total polyphenol content (TPC) was determined spectrophotometrically with Follin–Ciocalteu reagent and gallic acid as standard [81]. The results were read using spectrophotometer (Helios Gamma, Thermo Spectronic) at 760 nm. TPC was expressed as gallic acid equivalents (GAE) in mg 100 g^−1^ FW. 

##### Determination of Antioxidant Activity by 2,2-Diphenyl-1-picrylhydrazyl (DPPH) Free Radical Reduction

Leaf antioxidant activity was determined by a reduction of free radicals (DPPH), according to Yen and Chen [82], and DPPH inhibition percentage was calculated according to the formula provided by Rossi et al. [83]. Antioxidant potential of the investigated solutions was expressed by the percentage of DPPH inhibition using the following equation:

% of DPPH inhibition = 100 − [(At/Ar) × 100],(1)

where

At—absorbance of the test solution
Ar—absorbance of the reference solution

##### Determination of Na and Cl

To determine leaf content of Na and Cl, plant material was dried (80 °C for 48 h), pulverized and wet mineralized with 17 cm^3^ of concentrated H_2_SO_4_ (d = 1.84) to obtain a 2.0 g air-dried sample. Sodium levels were assessed using a flame photometer AFP-100 (Biotech Engineering Management, Nicosia, Cyprus). Chlorine determination involved quantitative precipitation of chlorides in a neutralized solution by titration with AgNO_3_ in the presence of K_2_CrO_4_ as an indicator [84].

##### Data Analysis

Both experiments were run as univariate ones in a complete randomization arrangement. All determinations were repeated four times. The measurements were statistically verified using the analysis of variance and Statistica 13 software (Statsoft, Cracov, Poland). Confidence intervals were calculated with Tukey’s test (*p* ≤ 0.05).

## 4. Conclusions

Sodium alginate is a compound with versatile applications and is completely safe for the environment. In horticulture and agriculture, it is a popular polymer used for encapsulation of seeds and plant tissues in micropropagation. Sodium alginate derivatives exert a biostimulatory effect on plant growth and development and may cause plant resistance to pathogens and stress. Our study showed that low molecular mass oligo-alginate used for bulb coating may be a potential candidate as a bioregulator in the cultivation of *Eucomis autumnalis*. The biostimulating effect was the most pronounced for the oligo-alginate of molecular mass 32,000 g mol^−1^. Plants treated with this oligomeric fraction were of markedly better quality, their leaves contained more pigments and total polyphenols and they exhibited slightly increased antioxidant activity. Also, our study was the first that showed that oligo-alginate may be used to improve plant tolerance to salt stress. The oligomer limited biomass loss, increased leaf content of photosynthetic pigments and curbed accumulation of sodium and chlorine in NaCl treated plants. These outcomes may help optimize the production technology of *Eucomis autumnalis* and possibly other bulbous plants. However, we need to remember that the effects of biostimulators may be different under controlled vs. field conditions, and the differences are due to the interaction of many factors, such as climatic conditions or soil properties. The mechanism of action of sodium alginate derivatives is still being researched. Further studies shall explore the relationships between the oligomer effectiveness and plant genotype, physical and chemical properties of the compound, its concentration or application method.

## Figures and Tables

**Table 1:** Effect of oligo-alginate with different molecular mass on growth and flowering of *Eucomis autumnalis*. Means (±standard errors) followed by the same small letter in the same row did not differ by Tukey test (*p* ≤ 0.05).

Parameters	Oligo-Alginate Molecular Mass (g mol^−1^)			
	Control	32,000	42,000	64,000
Days to anthesis	81.6 ± 0.78 a	75.2 ± 0.61 b	76.0 ± 0.50 b	77.2 ± 0.52 b
Plant height (cm)	31.2 ± 1.59 c	40.8 ± 1.01 a	37.5 ± 1.04 a,b	34.3 ± 1.64 b,c
Plant width (cm)	28.3 ± 1.04 b	36.4 ± 1.68 a	32.0 ± 1.80 a,b	32.5 ± 0.76 a,b
Leaf length (cm)	23.7 ± 1.29 b	32.0 ± 1.99 a	30.3 ± 0.79 a	32.4 ± 1.84 a
Number of leaves	5.33 ± 0.17 a	5.50 ± 0.29 a	5.50 ± 0.29 a	5.50 ± 0.29 a
Number of inflorescences	1.10 ± 0.06 a	1.10 ± 0.03 a	1.08 ± 0.04 a	1.03 ± 0.58 a
Inflorescence length (cm)	15.3 ± 0.88 b	20.3 ± 0.33 a	19.8 ± 0.33 a	21.2 ± 1.36 a
Number of florets	68.6 ± 4.03 c	88.7 ± 2.23 a	81.4 ± 1.99 b	81.4 ± 2.45 b

**Table 2:** Effect of oligo-alginate and NaCl on photosynthetic pigment concentrations in leaves of *Eucomis autumnalis*. Means (±standard errors) followed by the same small letter in the same row did not differ by Tukey test (*p* ≤ 0.05).

Parameters	Treatment			
	Control	Oligo-Alginate	NaCl	Oligo-Alginate + NaCl
Chlorophyll *a* (mg kg^−1^ FW)	434.2 ± 6.90 b	497.0 ± 5.50 a	342.3 ± 15.41 c	396.0 ± 4.51 b
Chlorophyll *b* (mg kg^−1^ FW)	166.6 ± 3.91 a,b	186.8 ± 0.96 a	146.5 ± 8.29 b	160.3 ± 1.72 b
Chlorophyll *a*/*b* ratio	2.61 ± 0.06 a,b	2.66 ± 0.04 a	2.34 ± 0.03 c	2.47 ± 0.02 b,c
Chlorophyll *a* + *b* (mg kg^−1^ FW)	600.8 ± 9.24 b	683.8 ± 5.12 a	488.8 ± 23.66 c	556.3 ± 6.02 b
Carotenoid (mg kg^−1^ FW)	170.2 ± 5.68 a,b	187.8 ± 2.05 a	153.3 ± 9.22 b	176.1 ± 2.53 a,b
Chlorophyll/carotenoid ratio	3.54 ± 0.06 a	3.64 ± 0.01 a	3.19 ± 0.06 b	3.16 ± 0.03 b

**Table 3:** Effect of oligo-alginate and NaCl on content of total polyphenols, l-ascorbic acid and antioxidant activity (2,2-diphenyl-1-picrylhydrazyl (DPPH) free radical reduction) of *Eucomis autumnalis*. Means (±standard errors) followed by the same small letter in the same row did not differ by Tukey Test (*p* ≤ 0.05).

Parameters	Treatment			
	Control	Oligo-Alginate	NaCl	Oligo-Alginate + NaCl
Total polyphenols (mg GAE 100 g^−1^ FW)	39.6 ± 1.56 c	57.6 ± 1.19 a	46.4 ± 2.29 b	49.1 ± 1.75 b
l-ascorbic acid (mg 100 g^−1^ FW)	27.4 ± 1.49 c	42.6 ± 2.02 a	30.8 ± 2.27 b	36.0 ± 2.25 a,b
Antioxidant activity (% DPPH)	1.64 ± 0.11 d	2.94 ± 0.25 a	2.18 ± 0.09 c	2.41 ± 0.08 b
